# Post–13-Valent Pneumococcal Conjugate Vaccine Dynamics in Young Children of Serotypes Included in Candidate Extended-Spectrum Conjugate Vaccines 

**DOI:** 10.3201/eid2701.201178

**Published:** 2021-01

**Authors:** Shalom Ben-Shimol, Noga Givon-Lavi, Leore Kotler, Bart Adriaan van der Beek, David Greenberg, Ron Dagan

**Affiliations:** Ben-Gurion University of the Negev, Beer Sheva, Israel (S. Ben-Shimol, N. Givon-Lavi, L. Kotler, B.A. van der Beek, D. Greenberg, R. Dagan);; Soroka University Medical Center, Beer Sheva (S. Ben-Shimol, N. Givon-Lavi, L. Kotler, D. Greenberg)

**Keywords:** pneumococcal vaccine, vaccines, *Streptocococcus pneumoniae*, bacteria, respiratory infections, Israel, antimicrobial resistance

## Abstract

After worldwide implementation of 10-valent and 13-valent pneumococcal conjugate vaccines (PCV10/PCV13), a 20-valent PCV (PCV20) was developed. We assessed dynamics of non-PCV13 additional PCV20 serotypes (VT20–13), compared with all other non-VT20 serotypes, in children <2 years of age in late PCV13 (2015–2017) and early PCV (2009–2011) periods. Our prospective population-based multifaceted surveillance included isolates from carriage in healthy children, children requiring chest radiography for lower respiratory tract infections (LRTIs), and children with non-LRTI illness, as well as isolates from acute conjunctivitis, otitis media (OM), and invasive pneumococcal disease (IPD). After PCV13 implementation, VT20–13 increased disproportionally in OM, IPD, and carriage in LRTI. VT20–13/non-VT20 prevalence ratio range was 0.26–1.40. VT20–13 serotypes were more frequently antimicrobial-nonsusceptible than non-VT20 serotypes. The disproportionate increase of VT20–13 in respiratory infections and IPD points to their higher disease potential compared with all other non-VT20 as a group.

*Streptococcus pneumoniae* is a major cause of illness and death worldwide (*1*,*2*). It causes otitis media, sinusitis, pneumonia, and invasive pneumococcal diseases (IPD) (*1*).

Capsular polysaccharides are considered the most important virulence factor in *S. pneumoniae* (*3*). Currently, >95 capsular serotypes have been identified. Each serotype is distinguished by the chemical structure of its polysaccharides, serologic response, and other genetic characteristics (*4*). Pneumococcal capsule types are associated with pathogenic processes including complement deposition, inflammation, and binding to the C-type lectin of host phagocytes (*3*). Pneumococcal serotype appears to be important in determining colonization, disease development, and clinical phenotype. Indeed, in the time before pneumococcal conjugate vaccines (PCVs), a limited number of serotypes among the known >95 serotypes were responsible for >70% of all IPD in children worldwide (*5*).

The routine use of PCVs in children worldwide has led to a decline of vaccine serotype (VT) IPD, mucosal diseases, and nasopharyngeal carriage (*6*–*9*). However, after PCV implementation, in spite of a decrease in overall pneumococcal disease rates, carriage of and disease from non-PCV serotypes (NVT) increased (*5*). As of October 2020, licensed PCVs contain 7 to 13 serotypes (7-valent [PCV7], 10-valent [PCV10], and 13-valent [PCV13]). Efforts to develop extended-spectrum (higher valency) PCVs have led to the development of 15- and 20-valent PCVs (PCV15, PCV20), both currently in advanced stages of clinical studies. The experimental PCV20 includes, beyond the 13 serotypes of PCV13 (1, 3, 4, 5, 6A, 6B, 7F, 9V, 14, 19A, 19F, 18C, and 23F), the additional pneumococcal serotypes 8, 10A, 11A, 12F, 15B/C, 22F and 33F (*10*), of which 2 (serotypes 22F and 33F) are also contained in PCV15 (*11*). These additional PCV20 serotypes (VT20–13) have been increasingly observed in recent years as common IPD serotypes. However, data are scarce in regard to their relative role in other entities such as carriage and respiratory disease in young children.

We compared the proportion rate dynamics of the added PCV20 serotypes (VT20–13) with PCV13 (VT13) and remaining non-VT20 (NVT20) serotypes in nasopharyngeal carriage of healthy young children and in children with non–lower respiratory tract infections (LRTIs) (pediatric emergency room [PER] visits in which chest radiography was not done), lower respiratory diseases requiring chest radiography, IPD, and pneumococcal culture-positive acute conjunctivitis and otitis media (OM).

## Materials and Methods

### Study Design

Our data derive from several ongoing, population-based, active surveillance projects conducted by the Pediatric Infectious Disease Unit, Soroka University Medical Center (SUMC) during 2009–2017. We defined 2 periods: early PCV (2009–2011) and late PCV13 (2015–2017). SUMC is the only hospital in southern Israel providing healthcare to the entire region, which enables us to conduct population-based studies.

### Setting and Study Population

During the study period, the average total number of annual births in the Negev district in southern Israel was ≈15,000. Over 95% of the children in the district are born and receive medical services at the SUMC. In the Negev district, the Jewish and the Bedouin populations live side-by-side. The socioeconomic conditions and the lifestyles of the 2 groups differ, but both have access to the same medical services. The Jewish population, mainly urban, resembles developed populations, whereas the Bedouin population, formerly desert nomads in transition to a Western lifestyle, resembles developing populations, with a high occurrence of infectious diseases including pneumococcal disease and complex OM (*6**,**7**,**9*). Contact between the children of the 2 populations is rare. During the study period, ≈50% of children <2 years of age in southern Israel were Jews and 50% were Bedouins. Our data derived from 5 prospective active surveillance projects.

#### 1) Carriage in Healthy Children (Group 1; Community Setting, Southern Israel)

This study, initiated in 2011, included nasopharyngeal cultures obtained from healthy children <2 years of age who were brought to the maternal and child healthcare centers in southern Israel for vaccination. A nasopharyngeal swab specimen was obtained after parents gave written informed consent.

#### 2) Carriage during Illness (Groups 2 and 3; Hospital PER Setting, Southern Israel)

This study, initiated in 2009, included cultures obtained from the PER of SUMC. Each workday, healthcare workers obtained nasopharyngeal cultures from the first 4 Jewish and 4 Bedouin children who were <2 years of age, residents of the Negev, and brought to the PER for any reason (*9*). We defined 2 groups in that surveillance: carriage in non-LRTI (children seen at the PER for any disease except those necessitating chest radiography); and carriage in LRTI (children with LRTI from whom a chest radiography was obtained) (*6*).

#### 3) Conjunctivitis (Group 4; Both Community and Hospital Settings, Southern Israel)

The study population included children <24 months of age who were residents of the Negev region, received a diagnosis of acute conjunctivitis in a community clinic or at SUMC (either PER or hospitalized), and had a conjunctival culture sent to the Clinical and Microbiology Laboratory of the SUMC since 2009.

#### 4) Otitis Media (Group 5; Both Community and Hospital Settings, Southern Israel)

The study population included children <24 months of age who were residents of the Negev region and had OM judged to necessitate middle ear fluid (MEF) culture. Cultures were obtained by tympanocentesis or by swab of the external canal of children with acute (<7 days) spontaneous otorrhea. Most of the children had complex OM (nonresponsive, recurrent, spontaneous perforation, or chronic ear effusion) (*7*,*12*,*13*). Children found to have pneumococcal MEF isolates during 2009–2017 were included (*7*).

#### 5) IPD (Group 6; Both Community and Hospital Settings, Israel, Nationwide)

This nationwide study was conducted in all 27 medical centers routinely obtaining cerebrospinal fluid (CSF) and blood cultures from children <24 months old in Israel; sites included 26 hospitals and 1 outpatient health maintenance organization (*14*). This setting enabled us to cover all culture-confirmed IPD cases among the population of Israel and calculate national incidence. No CSF cultures and <1% of blood cultures were obtained outside these centers. Data in our study were for IPD episodes identified since 2009.

### PCV Introduction to the Israeli National Immunization Plan and Uptake

Israel implemented PCV7 and PCV13 vaccination during July 2009–November 2010 on a schedule of 2, 4, and 12 months; catch-up vaccines were also administered for PCV7 in children <2 years. Vaccine uptake evaluation methods were as previously described (*14*). By June 2011, ≈80% of children 7–11 months of age had received >2 doses of PCV7, PCV13, or both, and ≈90% by December 2012; thereafter, ≈95% had received >2 PCV13 doses. By June 2011, a total of 36% of children 24–35 months of age had received >3 PCV7/PCV13 doses; that number increased to 87% by December 2012, and thereafter, >90% received >3 PCV13 doses.

### Bacteriology

#### Nasopharyngeal Cultures

Nasopharyngeal samples were obtained as previously described (*9*). In brief, we used a flexible Dacron-tipped swab, introduced through the nostrils. These swabs were inoculated into modified Stewart transport medium (Medical Wire and Equipment Co., Ltd, https://www.mwe.co.uk) and were processed within 16 hours at SUMC’s clinical microbiology laboratory. Material from swabs was plated on Columbia agar with 5% sheep blood and 5.0 mg/mL gentamicin and incubated for 48 h.

We presumptively identified *S. pneumoniae* on the basis of the presence of α-hemolysis and inhibition by optochin; we confirmed the identity of the bacteria present by a positive slide agglutination test result (Phadebact; MKL Diagnostics AB, http://www.mkldiagnostics.com).

#### Conjunctivitis Cultures

We enrolled in the study all patients <24 months of age who received a diagnosis of conjunctivitis from pediatricians at SUMC or at the primary clinical service in southern Israel and whose conjunctival swabs were cultured at SUMC’s clinical microbiology laboratory and grew *S. pneumoniae*. Swabbing methods were described previously (*15*). Specimen swabs were placed in transport medium and were processed in a similar manner to the nasopharyngeal swabs.

#### Otitis Media and Middle Ear Fluid Cultures

Specimen swabs were sent in transport medium. They were processed in a similar manner to the nasopharyngeal and conjunctival swabs.

#### IPD

Pneumococcal isolates from blood and CSF were initially identified by each center using local standard procedures as described previously (*14*,*16*).

### Serotypes

All strains were serotyped by Quellung reaction using the antisera of Statens Serum Institute, Copenhagen, Denmark. Methods of specimen transport were described previously (*7*,*9*,*14*).

### Antimicrobial Susceptibility Testing

We performed antimicrobial susceptibility testing by Etest (AB Biodisk, http://www.abbiodisk.com) and Kirby-Bauer disk diffusion in accordance with Clinical Laboratory Standards Institute recommendations (*17*). Antimicrobial nonsusceptibility among isolates was defined as MIC above or a zone diameter below the susceptibility breakpoint. For penicillin, we defined 2 nonsusceptibility cutoff values: MIC >0.1 mg/L and MIC >1.0 mg/L. We used 2 nonsusceptibility values because the low cutoff (MIC >0.1 mg/L) indicates nonsusceptibility to oral penicillin (used for mucosal infections) and parenteral penicillin for CNS infections, whereas the high MIC (MIC >1.0 mg/L) indicates the possibility of nonsusceptibility to parenteral penicillin for non-CNS infections. We used MIC >1.0 μg/mL for ceftriaxone. For all other antimicrobial drugs, we tested susceptibility by the Kirby-Bauer method: for erythromycin, a zone diameter of <21 mm; for trimethoprim/sulfamethoxazole, <19 mm; for tetracycline, <23 mm; for chloramphenicol, <20 mm; and for clindamycin, <19 mm. Isolates nonsusceptible to >3 of the antimicrobial categories were defined as multidrug resistant (MDR). In this study, we present antimicrobial susceptibility testing data for the late PCV13 period (2015–2017), for comparison of the current resistance patterns of VT20–13 versus the NVT20 serotype.

### Statistical Analysis

**Figure 2:**
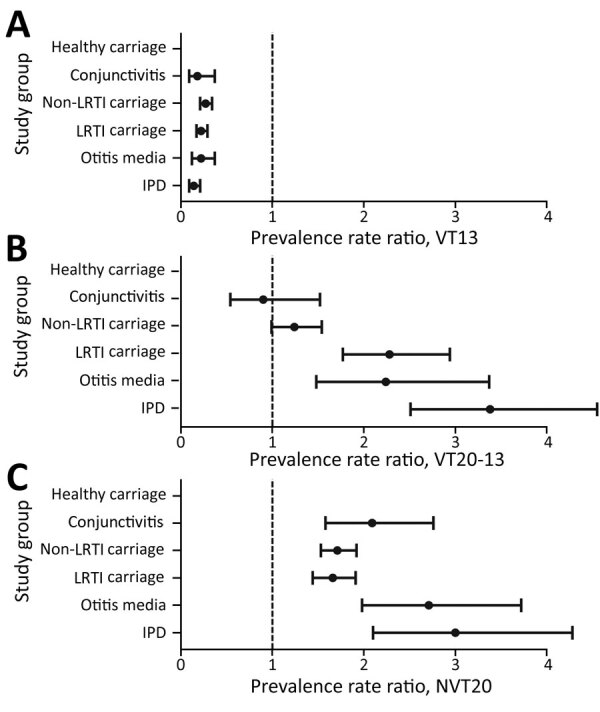
Prevalence rate ratios of pneumococcal VT13, VT20–13, and NVT20 of all pneumococcal isolates in carriage, conjunctivitis, OM, and IPD in children <24 months of age, Israel, comparing the late PCV13 period (2015–2017) to the early PCV period (2009–2011). The comparison could not be done for carriage in healthy children due to the nonavailability of data for the early PCV period. p<0.05 for all comparisons. Error bars indicate 95% CIs. IPD, invasive pneumococcal disease; LRTI, lower respiratory tract infection; NVT, nonvaccine serotype; OM, otitis media; PCV, pneumococcal conjugate vaccine; PCV13, 13-valent PCV; VT, vaccine serotype.

We conducted statistical analysis using SPSS Statistics 25.0 software for Windows (https://www.ibm.com/analytics/spss-statistics-software); p<0.05 was considered statistically significant. We analyzed data from active surveillance during 2009–2017 by epidemiologic years, July through June. We calculated prevalence rate ratios (RRs) with 95% CIs, comparing VT13, VT20–13, and NVT20 proportions in late PCV13 (2015–2017) versus early PCV (2009–2011) periods. Prevalence RRs were adjusted for age and ethnicity (Table 1; Figure 2).

**Table 1:** Postvaccine dynamics of pneumococcal conjugate vaccines in pediatric populations, Israel, October 2009–June 2017*

Epi year	Carriage in healthy children, n = 2,638				Carriage in non-LRTI, n = 2,450				Carriage in LRTI, n = 1,819				Conjunctivitis, n = 477				Otitis media, n = 756				IPD, n = 949		
	VT13	VT20–13	NVT20		VT13	VT20–13	NVT20		VT13	VT20–13	NVT20		VT13	VT20–13	NVT20		VT13	VT20–13	NVT20		VT13	VT20–13	NVT20
2009–10	ND	ND	ND		53	15	32		59	12	29		51	20	30		66	14	19		75	10	15
2010–11	ND	ND	ND		39	20	42		46	15	39		37	22	41		64	14	22		69	19	12
2011–12	21	15	64		26	21	53		29	19	51		28	20	52		36	22	42		35	48	17
2012–13	15	25	61		19	20	60		22	22	56		21	13	66		22	19	59		17	50	33
2013–14	10	25	65		15	20	65		19	16	65		14	23	62		23	28	49		19	53	28
2014–15	12	24	65		14	23	63		15	16	69		24	31	46		23	35	42		13	50	37
2015–16	11	24	66		10	23	67		14	32	56		13	17	70		16	27	57		9	52	39
2016–17	10	25	66		13	22	65		9	33	58		5	20	75		13	40	48		12	51	37
PRR†	NA‡				0.27 (0.21–0.34)	1.24 (0.99–1.54)	1.71 (1.53–1.92)		0.22 (0.17–0.29)	2.28 (1.77–2.94)	1.66 (1.44–1.91)		0.18 (0.09–0.37)	0.90 (0.54–1.52)	2.09 (1.58–2.76)		0.22 (0.12–0.37)	2.24 (1.48–3.37)	2.71 (1.98–3.72)		0.14 (0.09–0.21)	3.38 (2.51–4.55)	3.00 (2.10–4.28)

*Values are percentage of total pneumococcal isolates except as indicated. Epi, epidemiological; IPD, invasive pneumococcal disease; LRTI, lower respiratory tract infection; ND, no data; NVT20, serotypes not included in PCV20; PRR, prevalence rate ratio; VT13, serotypes included in 13-valent conjugate pneumococcal vaccine (PCV13); VT20-13, additional 20-valent PCV (PCV20) serotypes, not included in PCV13. †2015–2017 vs. 2009–11. ‡Data from 2009–2011 not available.

## Results

During the study period, a total of 9,089 isolates were analyzed: 2,638 from carriage in healthy children, 2,450 from carriage in non-LRTI, 1,819 from carriage in LRTI, 477 conjunctivitis, 756 OM, and 949 IPD isolates. Overall, throughout the study, 23.9% of all isolates were VT13, 23.3% were VT20–13, and 52.7% were NVT20 (Appendix Table 1).

### VT13, VT20–13 and NVT20 Proportions during the Early PCV Period

**Figure 1:**
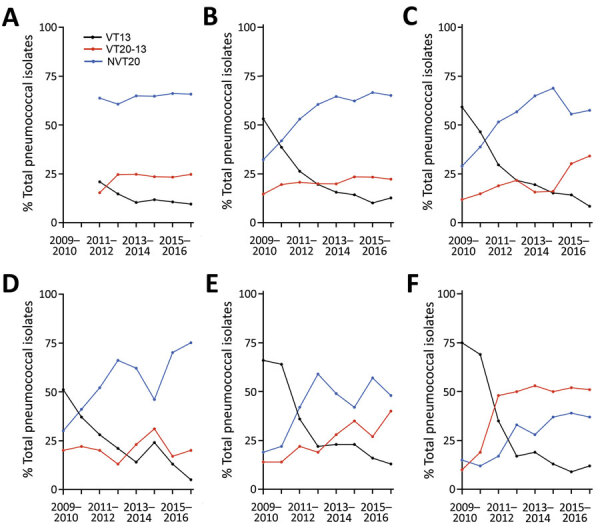
Postvaccine dynamics of pneumococcal conjugate vaccines in children <24 months of age, Israel, July 2009–June 2017. A) Healthy children; B) children with non–LRTIs; C) children with lower respiratory infections; D) children with conjunctivitis; E) children with otitis media (isolates from middle ear fluid were tested); and F) children with invasive pneumococcal disease (isolates from blood and cerebrospinal fluid were tested). Data show VT13, VT20–13, and NVT20 as the proportion of each serotype of all pneumococcal isolates. p<0.05 for all comparisons comparing 2015–2017 versus 2009–2011, except for VT20–13 in conjunctivitis. LRTI, lower respiratory tract infection; NVT, nonvaccine serotype; VT, vaccine serotype.

To enable better appreciation of disease potential of the 3 serotype groups (VT13, VT20–13 and NVT20) before PCV13 implementation, we analyzed the early PCV period separately. In the early PCV period, VT13 predominated in all groups. The proportions of VT13 of all isolates were 43.9% in carriage in non-LRTI, 52.1% carriage in LRTI, 45.1% conjunctivitis, 65% OM, and 71.8% IPD. Data for carriage in healthy children in this period were not available (Table 1; Figure 1).

For VT20–13, proportions were 17.8% for carriage in non-LRI, 13.4% in carriage in LRI, 20.5% conjunctivitis, 14.1% OM, and 14.8% IPD. For NVT20, proportions were 38.4% in carriage in non-LRI, 34.4% carriage in LRI, 34.4% for conjunctivitis, 20.9% OM, and 13.4% IPD.

### Proportion Dynamics of VT13, VT20–13, and NVT20 Comparing Late PCV13 Period with Early PCV Period 

The proportions of VT13 of all isolates declined significantly by 73%–86% in all groups when comparing late-PCV13 period with early-PCV period. During the late-PCV13 period, VT13 were identified in only 9%–14% of all pneumococcal isolates in the various groups (Figures 1, 2).

Proportions of VT20–13 did not increase significantly in conjunctivitis and carriage in non-LRI illness (Table 1; Figure 2). In contrast, we observed a significant increase of carriage in LRI, OM, and IPD (p<0.05 by test for trend in proportion for all groups). During the last study year, the proportions of VT20–13 of all identified pneumococcal isolates were 20%–25% in carriage in healthy children, carriage in non-LRTI, and conjunctivitis; 33% in carriage in LRTI; 40% in carriage in OM; and 51% in IPD. The fraction of VT20–13 became the leading one in IPD during the late-PCV13 period. The proportion rate ratios (2015–2017 vs. 2009–2011) for carriage in LRI, OM, and IPD were similar, whereas ratios for all 3 were significantly higher than those for conjunctivitis and carriage in non-LRTI disease.

Proportions of non-NVT20 increased in all groups, ranging from 66%–200% increase. The increase in IPD was significantly higher than in carriage in non-LRI and carriage in LRTI, but other increases did not differ significantly (Figure 2).

### VT20–13/NVT20 Ratio

**Figure 3:**
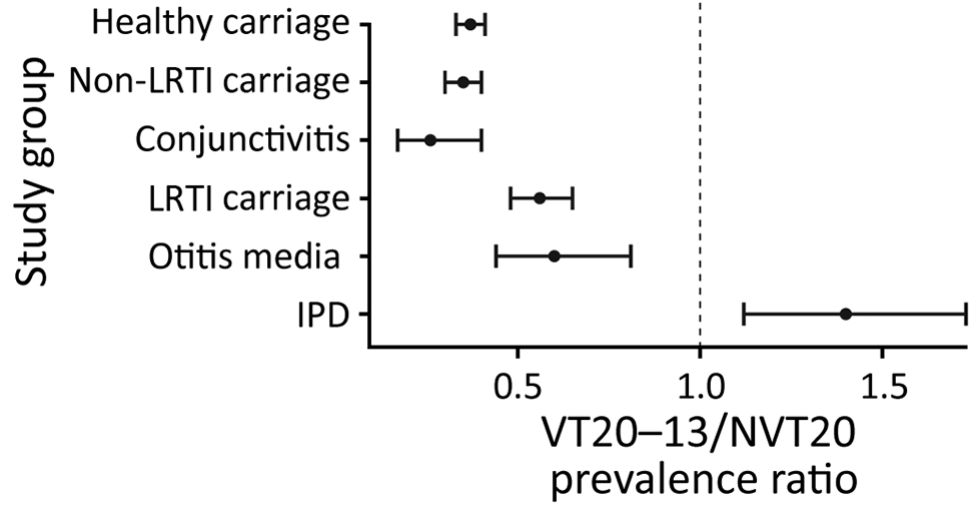
Ratio of prevalence of pneumococcal VT20–13/NVT20 ratio in children <24 months of age, Israel, during the late PCV13 period (2015–2017). Error bars represent 95% CI. IPD, invasive pneumococcal disease; LRTI, lower respiratory tract infection; NVT, nonvaccine serotype; PCV, pneumococcal conjugate vaccine; PCV13, 13-valent PCV; VT, vaccine serotype.

To assess the relative role of VT20–13 among all NVT, we calculated the ratio between VT20–13 and NVT20. In the early PCV period, VT20–13/NVT20 ratio was the lowest for carriage in LRTI (0.39) followed by carriage in non-LRTI (0.46), conjunctivitis (0.60), OM (0.68), and IPD (1.19) (Table 2). In the late-PCV13 period (2015–2017), VT20–13/NVT20 ratio was the lowest in conjunctivitis (0.26), followed by carriage in healthy children (0.37) and carriage in non-LRTI diseases (0.35) (Figure 3). The ratios for OM and carriage in LRTI were significantly higher than for the conjunctivitis, carriage in non-LRTI, and carriage in healthy children groups. The highest ratio was observed for IPD (1.40), significantly higher than all other outcomes.

**Table 2:** Proportions of VT20–13/NVT20 ratios in children age <24 mo, Israel, 2009–2011 and 2015–2017*

Clinical characteristic	2009–2011				2015–2017				2015–2017 vs. 2009–2011
	VT20–13, %	NVT20, %	VT20–13/NVT20 ratio (95% CI)		VT20–13, %	NVT20, %	VT20–13/NVT20 ratio (95% CI)		VT20–13/NVT20 ratio (95% CI)
Carriage in healthy children	NA	NA	NA		24	66	0.37 (0.33–0.41)		NA
Carriage in non-LRTI	18	38	0.46 (0.38–0.56)		23	66	0.35 (0.30–0.40)		0.80 (0.65–0.98)
Conjunctivitis	20	34	0.60 (0.39–0.91)		19	72	0.26 (0.17–0.40)		0.57 (0.35–0.94)
Carriage in LRTI	13	34	0.39 (0.30–0.50)		32	57	0.56 (0.48–0.65)		1.26 (1.00–1.60)
OM	14	21	0.68 (0.49–0.94)		34	52	0.60 (0.44–0.81)		0.91 (0.66–1.25)
IPD	15	13	1.19 (0.79–1.80)		52	38	1.40 (1.12–1.73)		1.05 (0.84–1.31)

*No data were available for healthy children in 2009–2011. IPD, invasive pneumococcal disease; LRTI, lower respiratory tract infection; NA, not applicable; NVT20, serotypes not included in PCV20; OM, otitis media; PCV, pneumococcal conjugate vaccine; VT20–13, additional 20-valent PCV (PCV20) serotypes, not included in PCV13.

### Serotype-Specific VT20–13 Dynamics

**Figure 4:**
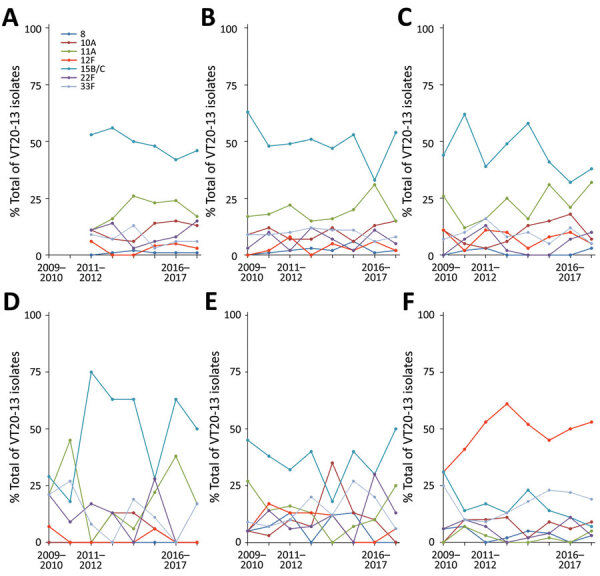
Serotype-specific VT20–13 pneumococcal isolates in children <24 months of age, Israel, 2009–2017. A) Healthy children; B) children with non–lower respiratory tract infections; C) children with lower respiratory tract infections; D) children with conjunctivitis; E) children with otitis media (isolates from middle ear fluid were tested); and F) children with invasive pneumococcal disease (isolates from blood and cerebrospinal fluid were tested). VT, vaccine serotype.

Of all 2,126 VT20–13 isolates, serotype 15B/C was the most common (40.7%), followed by serotypes 11A (16.9%), 12F (12.8%), 33F (10.4%), 10A (9.6%), 22F (7.6%), and 8 (2.0%). (Figure 4; Appendix Table 2). Of note, serotype 15B/C was the most common VT20–13 serotype in all clinical syndromes; it constituted 37%–49% of all VT20–13 in each group, except for IPD, in which serotype 12F was the most common (50% of all VT20–13).

### Antimicrobial Nonsusceptibility Rates among VT20–13 and NVT20 during the Late PCV13 Period

**Figure 5:**
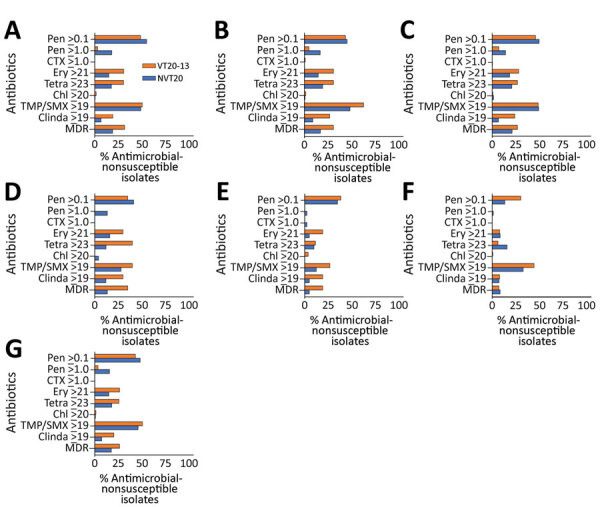
Proportion of antimicrobial drug–nonsusceptible isolates of all pneumococcal isolates in VT20–13 versus non-VT20 pneumococcal isolates in the late PCV13 period (2015–2017) in children <24 months of age in southern Israel. A) Healthy children; B) children with non–lower respiratory tract infections; C) children with lower respiratory tract infections; D) children with conjunctivitis; E) children with otitis media (isolates from middle ear fluid were tested); F) children with invasive pneumococcal disease; and G) all children. Chl, chloramphenicol; Clinda, clindamycin; CTX, ceftriaxone; Ery, erythromycin; MDR, multidrug resistance; PCV, pneumococcal conjugate vaccine; PCV13, 13-valent PCV; Pen, penicillin; Tetra, tetracycline; TMP/SMX, trimethoprim and sulphametoxazole; VT, vaccine serotype.

Antibiotic nonsusceptibility was higher among VT20–13 as a group than NVT20 for erythromycin, tetracycline, trimethoprim-sulfamethoxazole, clindamycin, and for MDR (Table 3; Figure 5). Similarly, in IPD, proportions of isolates with penicillin nonsusceptibility (MIC >0.1 µg/mL) were higher in VT20–13 compared with NVT20. In contrast, penicillin nonsusceptibility (MIC >0.1 µg/mL) was more prevalent in NVT20 than in VT20–13 in the all outcomes group; for penicillin nonsusceptibility with MIC ≥1.0 µg/mL, we observed these trends in carriage in healthy children, carriage in non-LRTI, and carriage in LRTI.

**Table 3:** Antimicrobial nonsusceptibility among pneumococcal isolates in children age <24 mo, Israel, 2015–2017*

Drug and MIC	Carriage in healthy children				Carriage in non-LRTI				Carriage in LRTI				Conjunctivitis				Otitis media				IPD				All outcomes		
	VT20-13, n = 261	NVT20, n = 706	p value		VT20–13, n = 150	NVT20, n = 433	p value		VT20–13, n = 141	NVT20, n = 253	p value		VT2–13, n = 20	NVT20, n = 75	p value		VT2–13, n = 26	NVT20, n = 40	p value		VT20–13, n = 112	NVT20, n = 82	p value		VT20–13, n = 710	NVT20, n = 1,589	p value
Penicillin >0.1	49	55	0.08		43	45	0.7		46	50	0.53		35	41	0.8		38	35	0.8		30	13	0.006		43	48	0.03
Penicillin >1.0	3	18	<0.001		5	17	<0.001		7	14	0.03		0	13	0.11		0	3	1.0		0	1	0.42		4	16	<0.001
Ceftriaxone >1.0	0	0	1.0		1	0	0.26		0	0	1.0		0	0	1.0		0	3	1.0		0	0	1.0		0	0	1.0
Erythromycin >21	31	15	<0.001		31	15	<0.001		28	19	0.03		30	16	0.2		19	5	0.1		8	9	1.0		26	15	<0.001
Tetracycline >23	31	18	<0.001		31	19	0.004		27	21	0.21		40	12	0.007		12	10	1.0		6	16	0.03		26	18	<0.001
Chloramphenicol >20	2	0	0.09		1	0	0.27		1	2	0.66		0	4	1.0		4	0	0.39		1	0	1.0		1	1	1.0
SXT >19	51	49	0.66		63	48	0.002		49	49	1.0		40	28	0.41		27	13	0.19		45	33	0.11		51	46	0.04
Clindamycin >19	19	7	<0.001		27	9	<0.001		24	7	<0.001		30	12	0.08		19	5	0.1		8	7	1.0		20	7	<0.001
MDR	32	19	<0.001		31	17	<0.001		27	21	0.22		35	13%	0.04		19	5	0.1		7	9	0.79		26	18	<0.001

*Values are percentage of total pneumococcal isolates except as indicated. IPD, invasive pneumococcal disease; LRTI, lower respiratory tract infection; MDR, multidrug resistance; NVT20, serotypes not included in PCV20; SXT, trimethoprim/sulfamethoxazole; VT20–13, additional 20-valent PCV (PCV20) serotypes, not included in 13-valent pneumococcal conjugate vaccine (PCV13).

## Discussion

Widespread implementation of PCVs has universally resulted in complete or near-complete nasopharyngeal colonization replacement of VT by NVT (*9*,*18*,*19*). This, in turn, resulted in increased disease by NVT to a variable extent, depending mainly on the disease entity (i.e., mucosal or invasive diseases) and environmental and host factors (*3*,*4*). The currently licensed PCVs contain <13 serotypes, and the remaining >85 serotypes constitute potential replacing serotypes. However, not all serotypes are equally capable of progressing from colonization to disease, and not all are equally successful colonizers, resulting in selection of specific serotypes that will eventually be frequently associated with disease. Furthermore, the most successful colonizers will likely increasingly become nonsusceptible to the commonly used antimicrobial drugs (*20*–*22*). Therefore, although invasive and mucosal pneumococcal disease are invariably reduced in children after PCV implementation, the remaining potential disease may be related disproportionally to a relatively small fraction of the remaining serotypes. In this study, we have shown that as a group, the 7 serotypes selected to be included in PCV20 are indeed disproportionally associated with disease and were frequently more drug-nonsusceptible than the other remaining NVT20. Although VT20–13/NVT20 ratios declined throughout the study for carriage in non-LRTI and conjunctivitis, these ratios increased or remained stable for carriage in LRTI, OM, and IPD. These trends point to the high disease potential of VT20–13 compared with NVT20 for IPD (in which VT20–13 caused >50% of all episodes in the last study period), and possibly for certain pneumococcal mucosal diseases such as OM and LRTI (in which VT20–13/NVT20 ratio increased, but NVT20 caused >50% of all episodes). We believe that these trends were driven by the vaccine-induced near-disappearance of the generally invasive PCV13 serotypes and the gradual differential replacement of those serotypes by NVT in different clinical syndromes, according to the specific NVT disease potential.

Several of the 7 additional PCV20 serotypes have been previously shown to have a high disease potential for IPD and other pneumococcal diseases (*4*,*5*,*23*–*26*). Nevertheless, all 7 serotypes were recognized as causing diseases and therefore were included in the 23-valent polysaccharide vaccine (PPV23), commonly used for the prevention of IPD in adults and in children >2 years of age with immunodeficiency and certain chronic medical conditions (*4*,*27*,*28*). Specifically, serotype 12F emerged as a major serotype in multiple countries, causing IPD and pneumonia (*5*,*10*,*22*–*24*,*29*,*30*). Its expansion was multiclonal, excluding a single hyperinvasive clone outbreak (*26*). This pattern bears similarities with the expansion of serotype 7F that occurred following PCV7 implementation; both serotypes were not expected to increase because they were frequently associated with low carriage. Serotype 8 was relatively uncommon in the current study but has emerged as one of the most prominent replacing serotypes in IPD in adults after PCV implementation, including in Israel (*23*–*25*,*31*). However, reports of this serotype in children are also emerging (*26*). Serotypes 22F and 33F, which are also included in an experimental 15-valent PCV currently in an advanced stage of clinical studies (*11*,*32*,*33*), were recognized as important serotypes in IPD and pneumonia (*11*,*24*,*25*,*34*). In the United States, proportions of IPD caused by serotype 22F were 11% in children <5 years and 13% in adults >18 years, whereas serotype 33F caused 10% of residual IPD cases in children <5 years and 5% in adults >18 years (*11*,*32*). In addition, serotypes 10A, 11A, and 15B/C, considered to have lower disease potential than serotypes 12F, 8, 22F, and 33F, have been reported frequently following PCV implementation worldwide (*5*,*10*,*23*–*25*).

Despite the importance of VT20 as a group, several NVT20 have been shown to be able to have the potential to cause large outbreaks (i.e., pneumococcal serotype 2 responsible for large IPD outbreaks in Israel) (*35*) or reported as emerging IPD-causing serotypes (i.e., serotype 24F) (*5*,*6*,*22*). Others, considered less invasive NVT20 serotypes have been also reported in several sites (i.e., serotypes 15A and 16F) (*36*–*38*).

This study adds important information not only on the proportions of specific serotypes causing IPD in the PCV13 era, but also with regard to the proportions of specific serotypes in carriage in healthy children versus children with IPD and mucosal diseases. These data are relevant because carriage during disease probably reflects relative disease potential in the various clinical entities. We found that both the rates of incidence and the relative proportion of VT20–13 seen in IPD, OM, and carriage in LRTI requiring chest radiography were significantly higher than those in conjunctivitis and in carriage in non-LRTI not requiring chest radiography. Specifically, in IPD, serotype 12F emerged as the most common serotype causing IPD in children <2 years of age, with ≈25% of all IPD caused by this serotype in recent years. Thus, the addition of serotype 12F coverage in PCV20 is expected to further decrease overall IPD rates in this population. Similarly, serotype 15B/C was the most common serotype in pneumococcal carriage and mucosal diseases (≈10% of all pneumococcal isolates).

We found that antimicrobial nonsusceptibility was significantly more frequent among VT20–13 than among NVT20; other studies have also shown such increases among PCV20–13 serotypes (*21*,*22*,*39*,*40*). In the pre-PCV era, most nonsusceptible strains belonged to serotypes included in PCV7/PCV13. Although nonsusceptibility is still higher among those strains or even increasing, the overall nonsusceptibility has been reduced in countries which successfully implemented PCVs (*21*,*22*,*39*). However, high selective pressure continues to occur with excessive antibiotic consumption, especially in dominating carriage serotypes. The most successful replacing serotypes are now those frequently exposed to antimicrobial drugs during carriage, resulting in increased antimicrobial nonsusceptibility compared with the less successful colonizers often found among NVT20 (*21*,*22*,*39*,*40*). Of interest, isolates causing noninvasive disease tend to have higher rates of antimicrobial resistance than those causing IPD (*21*), similar to the observations in our study.

The main strengths of our study include relatively long-term surveillance duration, large number of episodes, and the ability to assess multiple clinical outcomes in the same populations. Nonetheless, our study has several limitations. First, we did not have data on the pre-PCV period. However, we still could follow the dynamics of the non-PCV7/PCV13 serotypes because the data from the first year of implementation are included. Second, with regard to carriage in healthy children, data are only available from 2011–2012. However, the similarity of the proportion rates for 2010–2011 when carriage in healthy children is compared with that of children with non-LRTI and children with conjunctivitis (the most superficial mucosal disease, potentially explaining the higher proportion of noninvasive serotypes in the disease compared with other diseases) lends support to the suggestion that there are similarities between these groups and healthy children during the entire study period. Third, our data derive from multiple studies in the setting of a single country. However, as discussed above, similarities with other reports from other countries suggest that our conclusion may be generalized, at least to some extent.

In conclusion, *S. pneumoniae* VT20–13 are disproportionally associated with IPD, OM, and carriage in LRTI, compared with the other remaining NVT20, suggesting higher disease potential for these diseases than NVT20. In addition, the VT20–13 serotypes were more often nonsusceptible to various antimicrobial drugs than the NVT20 group. These findings suggest that PCV20 introduction may result in substantial decrease in the rates of IPD, OM, and possibly LRTI, as well as antimicrobial nonsusceptibility in children.

Appendix Additional information about postvaccine dynamics in candidate extended-spectrum 13-valent pneumococcal conjugate vaccines.

## References

[1] O’Brien KL, Wolfson LJ, Watt JP, Henkle E, Deloria-Knoll M, McCall N, et al.; Hib and Pneumococcal Global Burden of Disease Study Team. Burden of disease caused by *Streptococcus pneumoniae* in children younger than 5 years: global estimates. Lancet. 2009;374:893–902. 10.1016/S0140-6736(09)61204-6 19748398

[2] Wahl B, O’Brien KL, Greenbaum A, Majumder A, Liu L, Chu Y, et al. Burden of *Streptococcus pneumoniae* and *Haemophilus influenzae* type b disease in children in the era of conjugate vaccines: global, regional, and national estimates for 2000-15. Lancet Glob Health. 2018;6:e744–57. 10.1016/S2214-109X(18)30247-X 29903376 PMC6005122

[3] Jedrzejas MJ. Pneumococcal virulence factors: structure and function. Microbiol Mol Biol Rev. 2001;65:187–207. 10.1128/MMBR.65.2.187-207.2001 11381099 PMC99024

[4] Bogaert D, Hermans PWM, Adrian PV, Rümke HC, de Groot R. Pneumococcal vaccines: an update on current strategies. Vaccine. 2004;22:2209–20. 10.1016/j.vaccine.2003.11.038 15149779

[5] Balsells E, Guillot L, Nair H, Kyaw MH. Serotype distribution of *Streptococcus pneumoniae* causing invasive disease in children in the post-PCV era: A systematic review and meta-analysis. PLoS One. 2017;12:e0177113. 10.1371/journal.pone.0177113 28486544 PMC5423631

[6] Ben-Shimol S, Dagan R, Givon-Lavi N, Avital D, Bar-Ziv J, Greenberg D. Use of chest radiography examination as a probe for pneumococcal conjugate vaccine impact on lower respiratory tract infections in young children. Clin Infect Dis. 2020;71:177–87. 10.1093/cid/ciz768 31414125

[7] Ben-Shimol S, Givon-Lavi N, Leibovitz E, Raiz S, Greenberg D, Dagan R. Near-elimination of otitis media caused by 13-valent pneumococcal conjugate vaccine (PCV) serotypes in southern Israel shortly after sequential introduction of 7-valent/13-valent PCV. Clin Infect Dis. 2014;59:1724–32. 10.1093/cid/ciu683 25159581

[8] de Oliveira LH, Camacho LAB, Coutinho ESF, Martinez-Silveira MS, Carvalho AF, Ruiz-Matus C, et al. Impact and effectiveness of 10 and 13-valent pneumococcal conjugate vaccines on hospitalization and mortality in children aged less than 5 years in Latin American countries: a systematic review. PLoS One. 2016;11:e0166736. 10.1371/journal.pone.0166736 27941979 PMC5152835

[9] Ben-Shimol S, Givon-Lavi N, Greenberg D, Dagan R. Pneumococcal nasopharyngeal carriage in children <5 years of age visiting the pediatric emergency room in relation to PCV7 and PCV13 introduction in southern Israel. Hum Vaccin Immunother. 2016;12:268–76. 10.1080/21645515.2015.1095414 26430921 PMC5049747

[10] Thompson A, Lamberth E, Severs J, Scully I, Tarabar S, Ginis J, et al. Phase 1 trial of a 20-valent pneumococcal conjugate vaccine in healthy adults. Vaccine. 2019;37:6201–7. 10.1016/j.vaccine.2019.08.048 31495592

[11] Rupp R, Hurley D, Grayson S, Li J, Nolan K, McFetridge RD, et al. A dose ranging study of 2 different formulations of 15-valent pneumococcal conjugate vaccine (PCV15) in healthy infants. Hum Vaccin Immunother. 2019;15:549–59. 10.1080/21645515.2019.1568159 30689507 PMC6988874

[12] Ben-Shimol S, Givon-Lavi N, Leibovitz E, Greenberg D, Dagan R. Studying PCV impact on clinical presentation of otitis media helps to understand its pathogenesis. Vaccine. 2019;37:1–6. 10.1016/j.vaccine.2018.11.054 30497832

[13] Ben-Shimol S, Givon-Lavi N, Leibovitz E, Raiz S, Greenberg D, Dagan R. Impact of widespread introduction of pneumococcal conjugate vaccines on pneumococcal and nonpneumococcal otitis media. Clin Infect Dis. 2016;63:611–8. 10.1093/cid/ciw347 27225239

[14] Ben-Shimol S, Greenberg D, Givon-Lavi N, Schlesinger Y, Somekh E, Aviner S, et al. Early impact of sequential introduction of 7-valent and 13-valent pneumococcal conjugate vaccine on IPD in Israeli children <5 years: An active prospective nationwide surveillance. Vaccine. 2014;32:3452–9.10 10.1016/j.vaccine.2014.03.065 24690148

[15] Buznach N, Dagan R, Greenberg D. Clinical and bacterial characteristics of acute bacterial conjunctivitis in children in the antibiotic resistance era. Pediatr Infect Dis J. 2005;24:823–8. 10.1097/01.inf.0000178066.24569.98 16148850

[16] Ben-Shimol S, Greenberg D, Givon-Lavi N, Elias N, Glikman D, Rubinstein U, et al.; Israeli Bacteremia and Meningitis Active Surveillance Group. Rapid reduction in invasive pneumococcal disease after introduction of PCV7 into the National Immunization Plan in Israel. Vaccine. 2012;30:6600–7. 10.1016/j.vaccine.2012.08.012 22939907

[17] Clinical and Laboratory Standards Institute. Performance standards for antimicrobial susceptibility testing: 28th edition informational supplement (M100-S28). Wayne (PA): The Institute; 2018.

[18] Weinberger DM, Malley R, Lipsitch M. Serotype replacement in disease after pneumococcal vaccination. Lancet. 2011;378:1962–73. 10.1016/S0140-6736(10)62225-8 21492929 PMC3256741

[19] Usuf E, Bottomley C, Bojang E, Cox I, Bojang A, Gladstone R, et al. Persistence of nasopharyngeal pneumococcal vaccine serotypes and increase of nonvaccine serotypes among vaccinated infants and their mothers 5 years after introduction of pneumococcal conjugate vaccine 13 in The Gambia. Clin Infect Dis. 2019;68:1512–21. 10.1093/cid/ciy726 30165376 PMC6481996

[20] Danino D, Givon-Lavi N, Ben-Shimol S, Greenberg D, Dagan R. Understanding the evolution of antibiotic-nonsusceptible pneumococcal nasopharyngeal colonization following pneumococcal conjugate vaccine implementation in young children. Clin Infect Dis. 2019;69:648–56. 10.1093/cid/ciy926 30371763

[21] Richter SS, Diekema DJ, Heilmann KP, Dohrn CL, Riahi F, Doern GV. Changes in pneumococcal serotypes and antimicrobial resistance after introduction of the 13-valent conjugate vaccine in the United States. Antimicrob Agents Chemother. 2014;58:6484–9. 10.1128/AAC.03344-14 25136018 PMC4249410

[22] Varon E, Cohen R, Béchet S, Doit C, Levy C. Invasive disease potential of pneumococci before and after the 13-valent pneumococcal conjugate vaccine implementation in children. Vaccine. 2015;33:6178–85. 10.1016/j.vaccine.2015.10.015 26476365

[23] Amin-Chowdhury Z, Iyanger N, Ramsay ME, Ladhani SN. Outbreaks of severe pneumococcal disease in closed settings in the conjugate vaccines era, 2010-2018: A systematic review to inform national guidance in the UK. J Infect. 2019;79:495–502. 10.1016/j.jinf.2019.10.009 31629865

[24] Pick H, Daniel P, Rodrigo C, Bewick T, Ashton D, Lawrence H, et al. Pneumococcal serotype trends, surveillance and risk factors in UK adult pneumonia, 2013-18. Thorax. 2020;75:38–49. 10.1136/thoraxjnl-2019-213725 31594801

[25] Levy C, Ouldali N, Caeymaex L, Angoulvant F, Varon E, Cohen R. Diversity of serotype replacement after pneumococcal conjugate vaccine implementation in Europe. J Pediatr. 2019;213:252–253.e3. 10.1016/j.jpeds.2019.07.057 31561776

[26] Lo SW, Gladstone RA, van Tonder AJ, Lees JA, du Plessis M, Benisty R, et al.; Global Pneumococcal Sequencing Consortium. Pneumococcal lineages associated with serotype replacement and antibiotic resistance in childhood invasive pneumococcal disease in the post-PCV13 era: an international whole-genome sequencing study. Lancet Infect Dis. 2019;19:759–69. 10.1016/S1473-3099(19)30297-X 31196809 PMC7641901

[27] Hausdorff WP, Feikin DR, Klugman KP. Epidemiological differences among pneumococcal serotypes. Lancet Infect Dis. 2005;5:83–93. 10.1016/S1473-3099(05)70083-9 15680778

[28] Djennad A, Ramsay ME, Pebody R, Fry NK, Sheppard C, Ladhani SN, et al. Effectiveness of 23-valent polysaccharide pneumococcal vaccine and changes in invasive pneumococcal disease incidence from 2000 to 2017 in those aged 65 and over in England and Wales. EClinicalMedicine. 2019;6:42–50. 10.1016/j.eclinm.2018.12.007 31193709 PMC6537583

[29] Rokney A, Ben-Shimol S, Korenman Z, Porat N, Gorodnitzky Z, Givon-Lavi N, et al. Emergence of *Streptococcus pneumoniae* serotype 12F after sequential introduction of 7- and 13-valent vaccines, Israel. Emerg Infect Dis. 2018;24:453–61. 10.3201/eid2403.170769 29460732 PMC5823333

[30] Cohen R, Levy C, Bonnet E, Thollot F, Boucherat M, Fritzell B, et al. Risk factors for serotype 19A carriage after introduction of 7-valent pneumococcal vaccination. BMC Infect Dis. 2011;11:95. 10.1186/1471-2334-11-95 21501471 PMC3101155

[31] Regev-Yochay G, Katzir M, Strahilevitz J, Rahav G, Finn T, Miron D, et al.; IAIPD group. The herd effects of infant PCV7/PCV13 sequential implementation on adult invasive pneumococcal disease, six years post implementation; a nationwide study in Israel. Vaccine. 2017;35:2449–56. 10.1016/j.vaccine.2017.03.031 28342668

[32] Moore MR, Link-Gelles R, Schaffner W, Lynfield R, Lexau C, Bennett NM, et al. Effect of use of 13-valent pneumococcal conjugate vaccine in children on invasive pneumococcal disease in children and adults in the USA: analysis of multisite, population-based surveillance. Lancet Infect Dis. 2015;15:301–9. 10.1016/S1473-3099(14)71081-3 25656600 PMC4876855

[33] Pilishvili T, Lexau C, Farley MM, Hadler J, Harrison LH, Bennett NM, et al.; Active Bacterial Core Surveillance/Emerging Infections Program Network. Sustained reductions in invasive pneumococcal disease in the era of conjugate vaccine. J Infect Dis. 2010;201:32–41. 10.1086/648593 19947881

[34] van Hoek AJ, Andrews N, Waight PA, George R, Miller E. Effect of serotype on focus and mortality of invasive pneumococcal disease: coverage of different vaccines and insight into non-vaccine serotypes. PLoS One. 2012;7:e39150. 10.1371/journal.pone.0039150 22815698 PMC3398022

[35] Dagan R. Ben-Shimol S, Benisty R, Regev- Yochay G, Ron M, Givon-Lavi N, et al. A nationwide outbreak of invasive pneumococcal disease (IPD) caused by a novel *Streptococcus pneumoniae* serotype 2 (SP2) clone in the PCV13 era, in Israel. Abstract 1888. In: Abstracts of IDweek 2019; October 2–6, 2019; Washington, DC, USA. Alexandria (VA): Infectious Diseases Society of America; 2019.

[36] Nakano S, Fujisawa T, Ito Y, Chang B, Matsumura Y, Yamamoto M, et al. Whole-genome sequencing analysis of multidrug-resistant serotype 15A *Streptococcus pneumoniae* in Japan and the emergence of a highly resistant serotype 15A-ST9084 clone. Antimicrob Agents Chemother. 2019;63:e02579–18. 10.1128/AAC.02579-18 30803976 PMC6496056

[37] Neves FPG, Cardoso NT, Cardoso CAA, Teixeira LM, Riley LW. Direct effect of the 13-valent pneumococcal conjugate vaccine use on pneumococcal colonization among children in Brazil. Vaccine. 2019;37:5265–9. 10.1016/j.vaccine.2019.07.056 31337592

[38] Dayie NTKD, Tettey EY, Newman MJ, Bannerman E, Donkor ES, Labi A-K, et al. Pneumococcal carriage among children under five in Accra, Ghana, five years after the introduction of pneumococcal conjugate vaccine. BMC Pediatr. 2019;19:316. 10.1186/s12887-019-1690-5 31488088 PMC6727402

[39] Cohen R, Biscardi S, Levy C. The multifaceted impact of pneumococcal conjugate vaccine implementation in children in France between 2001 to 2014. Hum Vaccin Immunother. 2016;12:277–84. 10.1080/21645515.2015.1116654 26905678 PMC5049719

[40] Tomczyk S, Lynfield R, Schaffner W, Reingold A, Miller L, Petit S, et al. Prevention of antibiotic-nonsusceptible invasive pneumococcal disease with the 13-valent pneumococcal conjugate vaccine. Clin Infect Dis. 2016;62:1119–25. 10.1093/cid/ciw067 26908787

