# Supplementary material for: Post–13-Valent Pneumococcal Conjugate Vaccine Dynamics in Young Children of Serotypes Included in Candidate Extended-Spectrum Conjugate Vaccines
Source: Emerg Infect Dis. 2021 Jan;27(1):150–60. doi: 10.3201/eid2701.201178 (PMC7774550; doi:10.3201/eid2701.201178)
Supplement: Appendix — Additional information about postvaccine dynamics in candidate extended-spectrum 13-valent pneumococcal conjugate vaccines. [file 20-1178-Techapp-s1.pdf]

Article DOI: https://doi.org/10.3201/eid2701.201178

Post–13-Valent Pneumococcal Conjugate Vaccine Dynamics in Young Children of Serotypes Included in Candidate Extended-Spectrum Conjugate Vaccines

Appendix

Appendix Table 1. Number of pneumococcal isolates from nasopharyngeal pneumococcal carriage, conjunctivitis, otitis media, and IPD (by serotype groups VT13, VT20–13 and NVT20 pneumococcal isolates) in children <24 mo, Israel, July 2009–June 2017*

	Carriage in healthy children n = 2,638			Carriage in non-LRI n = 2,450			Carriage in LRI n = 1,819			Conjunctivitis n = 477			Otitis media n = 756			IPD n = 949			Total, n = 9,089		
Epi year	VT13	VT20–13	NVT-20	VT13	VT20–13	NVT-20	VT13	VT20–13	NVT-20	VT13	VT20–13	NVT-20	VT13	VT20–13	NVT-20	VT13	VT20–13	NVT-20	VT13	VT20–13	NVT-20
2009–10	NA	NA	NA	127	35	77	136	27	67	36	14	21	96	21	28	101	13	20	496	110	213
2010–11	NA	NA	NA	162	82	176	132	42	110	19	11	21	134	29	46	103	29	18	550	193	371
2011–12	65	47	195	75	59	151	58	38	101	17	12	31	50	31	59	43	58	21	308	245	558
2012–13	61	102	251	56	59	175	51	51	132	13	8	41	18	15	48	16	46	30	215	281	677
2013–14	46	110	288	44	57	184	36	31	123	10	16	43	14	17	30	23	66	35	173	297	703
2014–15	46	94	257	39	64	172	36	39	164	14	18	27	10	15	18	14	56	41	159	286	679
2015–16	48	105	292	36	85	241	32	68	127	6	8	32	6	10	21	9	54	40	137	330	753
2016–17	61	156	414	37	65	192	19	73	126	3	12	44	5	16	19	13	58	42	138	380	837
Total	327	614	1697	576	506	1368	500	369	950	118	99	260	333	154	269	322	380	247	2176	2122	4791
	12.4%	23.3%	64.3%	23.5%	20.7%	55.8%	27.5%	20.3%	52.2%	24.7%	20.8%	54.5%	44.0%	20.4%	35.6%	33.9%	40.0%	26.0%	23.9%	23.3%	52.7%

*IPD, invasive pneumococcal disease; LRI, lower tract respiratory infection; NA, data not available; NVT20, serotypes not included in PCV20; VT13, serotypes included in 13-valent conjugate pneumococcal vaccine (PCV13); VT20–13, additional 20-valent PCV (PCV20) serotypes, not included in PCV13.

Appendix Table 2. Specific VT20–13 pneumococcal serotypes in children <24 mo, Israel, 2009– 2017*

		Serotype							
Clinical entity	Epi year	8	10A	11A	12F	15B/C	22F	33F	VT20–13
Carriage in healthy children	2009–10	NA	NA	NA	NA	NA	NA	NA	NA
	2010–11	NA	NA	NA	NA	NA	NA	NA	NA
	2011–12	0	5	5	3	25	5	4	47
	2012–13	1	7	16	0	57	14	7	102
	2013–14	2	7	29	0	55	3	14	110
	2014–15	1	13	22	4	45	6	3	94
	2015–16	1	16	25	5	44	8	6	105
	2016–17	1	20	26	5	72	23	9	156

Clinical entity	Epi year	Serotype 8	10A	11A	12F	15B/C	22F	33F	VT20–13
Carriage in non-LRI	2009–10	0	3	6	0	22	1	3	35
	2010–11	1	10	15	2	39	8	7	82
	2011–12	1	4	13	5	29	1	6	59
	2012–13	2	4	9	0	30	7	7	59
	2013–14	1	7	9	3	27	4	6	57
	2014–15	4	4	13	1	34	1	7	64
	2015–16	1	11	26	5	28	9	5	85
	2016–17	1	10	10	1	35	3	5	65
Carriage in LRI	2009–10	0	3	7	3	12	0	2	27
	2010–11	1	2	5	1	26	3	4	42
	2011–12	1	1	6	4	15	5	6	38
	2012–13	0	3	13	5	25	1	4	51
	2013–14	0	4	5	1	18	0	3	31
	2014–15	0	6	12	3	16	0	2	39
	2015–16	0	12	14	7	22	5	8	68
	2016–17	2	5	23	4	28	7	4	73
Conjunctivitis	2009–10	0	0	3	1	4	3	3	14
	2010–11	0	0	5	0	2	1	3	11
	2011–12	0	0	0	0	9	2	1	12
	2012–13	0	1	1	0	5	1	0	8
	2013–14	0	2	1	0	10	0	3	16
	2014–15	0	1	4	1	5	5	2	18
	2015–16	0	0	3	0	5	0	0	8
	2016–17	0	0	2	0	6	2	2	12
Otitis media	2009–10	1	1	6	1	10	1	2	22
	2010–11	2	1	4	5	11	4	2	29
	2011–12	4	3	5	4	10	2	3	31
	2012–13	0	1	2	2	6	1	3	15
	2013–14	2	6	0	2	3	2	2	17
	2014–15	2	2	1	0	6	0	4	15
	2015–16	0	1	1	0	3	3	2	10
	2016–17	0	0	4	1	8	2	1	16
IPD	2009–10	1	0	0	5	5	1	4	16
	2010–11	2	3	2	12	4	3	3	29
	2011–12	0	6	2	31	10	4	5	58
	2012–13	1	5	0	28	6	0	6	46
	2013–14	3	1	0	34	15	1	12	66
	2014–15	2	5	1	25	8	2	13	56
	2015–16	0	3	0	27	6	6	12	54
	2016–17	2	5	3	31	4	2	11	58

*IPD, invasive pneumococcal disease; LRI, lower tract respiratory infection; NVT20, serotypes not included in PCV20; VT20–13, additional 20-valent pneumococcal conjugate vaccine (PCV20) serotypes, not included in 13-valent PCV (PCV13).
